# No Immune Responses by the Expression of the Yeast Ndi1 Protein in Rats

**DOI:** 10.1371/journal.pone.0025910

**Published:** 2011-10-03

**Authors:** Mathieu Marella, Byoung Boo Seo, Terence R. Flotte, Akemi Matsuno-Yagi, Takao Yagi

**Affiliations:** 1 Department of Molecular and Experimental Medicine, The Scripps Research Institute, La Jolla, California, United States of America; 2 Gene Therapy Center and Department of Pediatrics, University of Massachusetts Medical School, Worcester, Massachusetts, United States of America; Instituto de Química - Universidade de São Paulo, Brazil

## Abstract

**Background:**

The rotenone-insensitive internal NADH-quinone oxidoreductase from yeast, Ndi1, has been shown to work as a replacement molecule for complex I in the respiratory chain of mammalian mitochondria. In the so-called transkingdom gene therapy, one major concern is the fact that the yeast protein is foreign in mammals. Long term expression of Ndi1 observed in rodents with no apparent damage to the target tissue was indicative of no action by the host's immune system.

**Methodology/Principal Findings:**

In the present study, we examined rat skeletal muscles expressing Ndi1 for possible signs of inflammatory or immune response. In parallel, we carried out delivery of the *GFP* gene using the same viral vector that was used for the *NDI1* gene. The tissues were subjected to H&E staining and immunohistochemical analyses using antibodies specific for markers, CD11b, CD3, CD4, and CD8. The data showed no detectable signs of an immune response with the tissues expressing Ndi1. In contrast, mild but distinctive positive reactions were observed in the tissues expressing GFP. This clear difference most likely comes from the difference in the location of the expressed protein. Ndi1 was localized to the mitochondria whereas GFP was in the cytosol.

**Conclusions/Significance:**

We demonstrated that Ndi1 expression did not trigger any inflammatory or immune response in rats. These results push forward the Ndi1-based molecular therapy and also expand the possibility of using foreign proteins that are directed to subcellular organelle such as mitochondria.

## Introduction

Defects in the mitochondrial NADH-quinone oxidoreductase (complex I) have been shown to lead to many human diseases [1], [2]. We have developed a gene therapy strategy that utilizes the *NDI1* gene encoding the yeast rotenone-insensitive internal NADH-quinone oxidoreductase (Ndi1) [2]–[4]. The principle of this approach is that the yeast Ndi1 enzyme can replace functionality of defective complex I in the respiratory chain of mammalian mitochondria. We showed that injection of recombinant adeno-associated virus (rAAV) carrying the *NDI1* gene into the brain and skeletal muscles of rats and mice resulted in functional expression of the transgene and that the expressed Ndi1 had protective effects against Parkinsonian symptoms in the rotenone-treated rats [5] and MPTP-treated mice [6]. More recent work involved the restoration of vision by delivering the *NDI1* gene into the superior colliculus of a rat animal model of Leber's hereditary optic neuropathy [7]. Clearly, Ndi1 acted as a member of the respiratory chain in the rodent mitochondria and was able to compensate for the malfunctioning complex I.

A potential obstacle to any gene therapy is possible development of inflammatory and/or an immune response caused by the vector or the transgene itself [8], [9]. In our case, we use recombinant adeno-associated virus (rAAV) as the vector which has a high safety profile as compared to other viral vectors [10]–[12]. However, our therapeutic molecule is a yeast protein which could initiate a severe immune response in mammals. For example, it has been reported that intravenous administration of foreign transgene products such as the *E. coli lacZ* gene and GFP cDNA induced severe liver injury in mice [13]. Because of this problem, the consensus is that self-genes should be used for the therapy. There is no guarantee even for a human gene to be immunologically safe if it was utilized for therapy of human disease. In a clinical trial of severe hemophilia B, an injection of human factor IX cDNA into the human hepatic artery could express factor IX but the therapeutic level in the blood was retained only for 8 weeks because of destruction of transduced hepatocytes by cell-mediated immunity [14]. In contrast, Ndi1 has been shown to persist over a long period of time. In rodents, we observed the presence of Ndi1 for at least 7 months [15], [16]. In *Drosophila*, this yeast protein survived throughout the entire life span [17]. It should be noted that, while the flies do not have an immunoglobulin-based immune response, their immune system is used as a model for the human innate immune response. These results led us to believe that, despite the fact that Ndi1 is a foreign protein, it should not pose immune-related problems in higher animals. It is therefore of interest to investigate the inflammatory and immune response of Ndi1-expressed organs in rodents not only to confirm the *in vivo* observations but also to gain insights into the reason for the apparent lack of immune problems.

## Results

### No inflammation was observed with Ndi1 expression in the muscle and the brain

**Figure 1:**
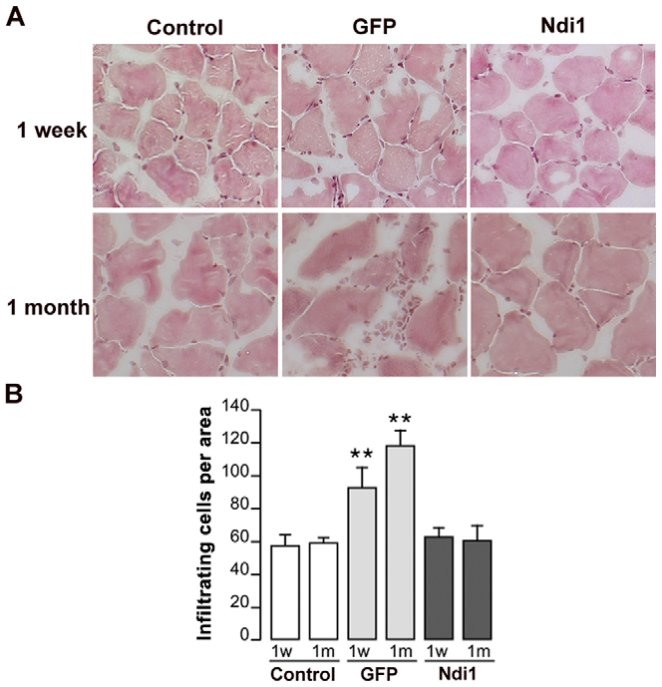
H&E staining of coronal sections of rat muscles. Rats were injected with either PBS, rAAV-GFP, or rAAV-NDI1 in the skeletal muscles. Muscles (tibialis anteriors) were collected at 1 week (1w) or 1 month (1 m) and were stained with Hematoxylin-Eosin (H&E) solutions and infiltrated cells were counted. (A) Representative images of muscle sections from each group after H&E staining. (B) Comparison of the number of infiltrated cells. The histogram shows the number of small mononuclear cells per field of view in the sections separated by at least 90 µm (n = 4 for rats, n = 6 for muscle sections from each animal). Results are expressed as mean ± SD. ** p<0.01 from the respective control (Student's t-test).

**Figure 2:**
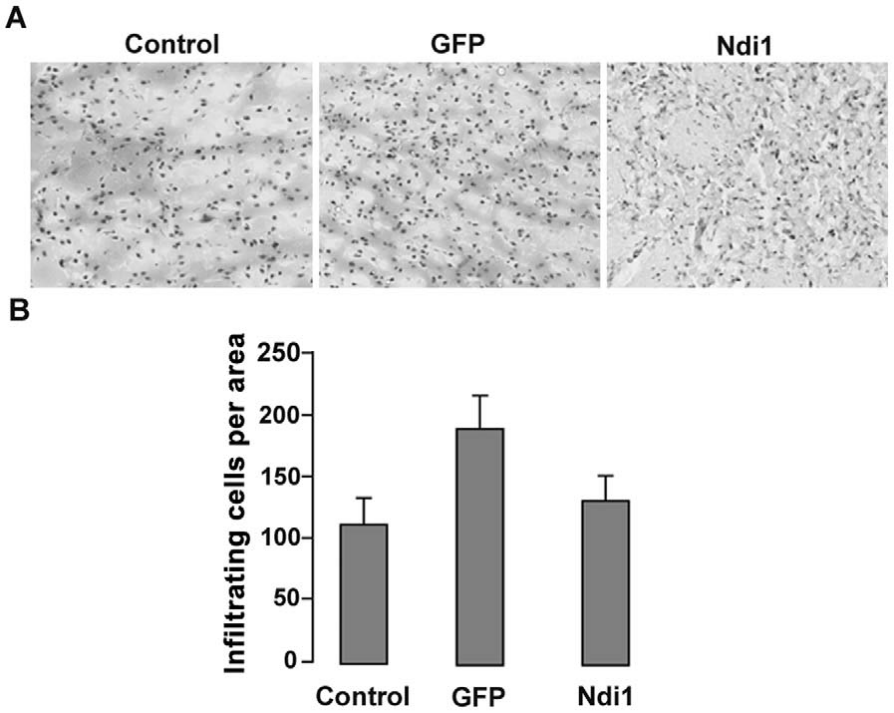
Evaluation of infiltrated cells in the rat brain. Rats were injected with PBS (control), rAAV-GFP, or rAAV-NDI1 in the striatum of rat brain. The brain samples were collected 1 month post-injection. (A) Representative images of brain sections from each group after H&E staining. (B) Comparison of the number of infiltrated cells. The histogram shows the number of infiltrating cells per field of view in the sections separated by at least 90 µm (n = 4 for rats, n = 6 for brain sections from each animal). Results are expressed as mean ± SD. ** p<0.01 and * p<0.05 from the respective control (Student's t-test).

Infiltration of cells in tissue is one of the most visible signs of inflammation and can be visualized by hematoxylin and eosin (H&E) staining. We did a single injection of rAAV carrying the *NDI1* gene (rAAV-NDI1) or the *GFP* gene (rAAV-GFP) into the rat skeletal muscle and examined the sections using H&E staining 1 week or 1 month post-injection. Figure 1A shows representative images of the staining. Analysis of tissue sections from rAAV-NDI1-injected muscles showed normal morphology without inflammatory infiltrate or tissue damage at 1 week as well as at 1 month. In contrast, muscle sections from the area expressing the GFP protein exhibited distinctly higher staining than control both in the 1-week and the 1-month samples. The number of infiltrating cells was counted for each group and compiled into histograms (Figure 1B). Activation of macrophages is apparent in the muscle tissue expressing GFP. On the other hand, no difference was observed between the muscle sections from the Ndi1 group and the control. We have performed a similar experiment using the striatum of the rat brain as a target tissue and evaluated the sections for signs of inflammation 1 month after a single injection of rAAV-NDI1 or rAAV-GFP (Figure 2A and 2B). The brain is considered to be an immunologically protected space [18]. This immune privilege is the result of multiple layers and mechanisms, including the blood-brain barrier. A main component of the immune system in brain is microglia that are derived from the circulating macrophages. Thus, the brain has active immune defense that shares the same principal characteristics [19]. In fact, an elevated number of infiltrating cells were clearly seen in the brain sections expressing GFP. Once again, Ndi1 expression did not elicit any inflammatory response.

### Absence of cytotoxic cellular responses against Ndi1

**Figure 3:**
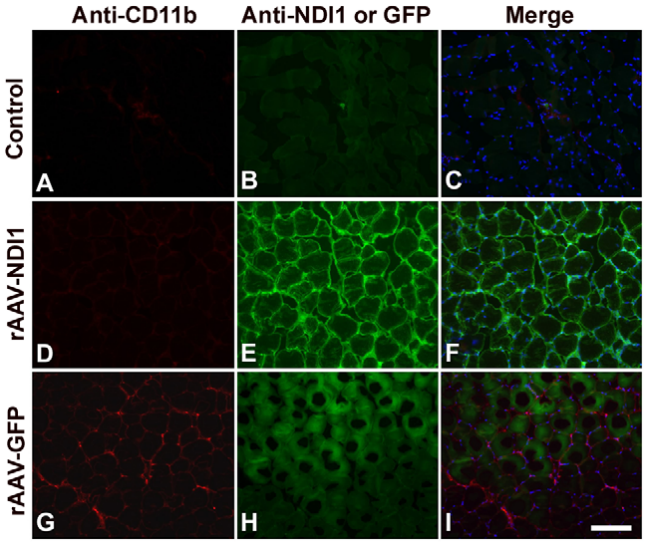
Representative images of macrophage staining of tissue sections from rat muscles. Rats were injected with PBS, rAAV-GFP, or rAAV-NDI1 in the skeletal muscles and muscle samples were collected 1 month post-injection. Coronal sections of the muscle from the animals injected with PBS (Control), rAAV-NDI1 or rAAV-GFP were stained with anti-CD11b antibody (A, D, and G, red) or with anti-Ndi1 antibody (E, green). Muscle sections from the rats injected with rAAV-GFP were examined for the fluorescence of the GFP protein (H, green). A green channel image for the control (B) is also shown. Merged images of the red and green channels are also shown (C, F, and I) Scale bar = 75 µm.

**Figure 4:**
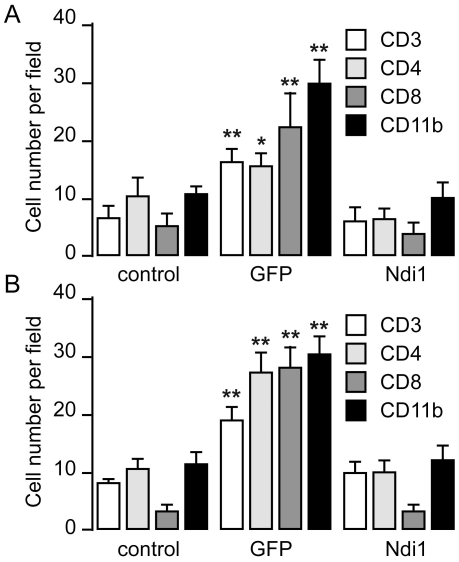
Immune cells distribution within the injected area of the rat muscle. Rats received an injection of rAAV-GFP or rAAV-NDI1 in the skeletal muscles. Muscle samples were harvested either 1 week (A) or 1 month (B) after the injection. Immunohistochemistry was performed on coronal sections of rat muscles expressing the GFP and the Ndi1 protein. Antibodies against immunological marker proteins, CD3, CD4, CD8, CD11b, were used and positively stained cells were counted (n = 4 for rats, n = 6 for brain sections from each animal). Results are expressed as mean ± SD. ** p<0.01 and * p<0.05 from the respective control (Student's t-test).

In addition to the H&E staining that allows for visualization of inflammation, we carried out immunohistochemistry experiments using specific antibodies. When the monocytes become activated, they express a cluster of differentiations such as CD11b. CD11b molecules are on the plasma membrane of microglia or macrophage monocyte antigen presenting cells. Representative images of immunohistochemical staining for CD11b and expressed Ndi1 are shown in Figure 3. Muscle tissue sections in which a high level of Ndi1 was observed showed totally negative staining for CD11b. Muscle sections from the rats that received a rAAV-GFP injection exhibited good GFP fluorescence. As expected, the GFP-expressing tissue was positive for the CD11b staining. The activated monocytes participate in the recruitment of the T-cell to the site of infection that will eliminate the pathogenic factors and infected cells. This recruitment can be directly followed by immunohistochemistry. To assess the adaptive immune response, we performed immunohistochemistry on the same samples using antibodies against CD3 (T cell marker), CD4 (helper T cell marker), and CD8 (cytotoxic T cell marker), Figure S1. The number of cells that were stained by the respective antibodies was counted and transformed into histograms (Figure 4). For all cellular markers tested, the expression of GFP led to a consistent increase in the population of marker-positive cells. The extent of increase varied from ∼2 fold for CD3-positive T cells to almost 9-fold for CD8-positive T cells in the samples collected 1 month after the delivery of the gene. The presence of these T lymphocytes was concomitant with the elevation of infiltrating cells. In the case of the muscle tissues from the Ndi1-expressing rats, there was no significant difference when compared with the PBS control in any of the markers examined. These results are in accordance with those obtained by H&E staining.

### Absence of humoral immune response against the Ndi1 protein

**Figure 5:**
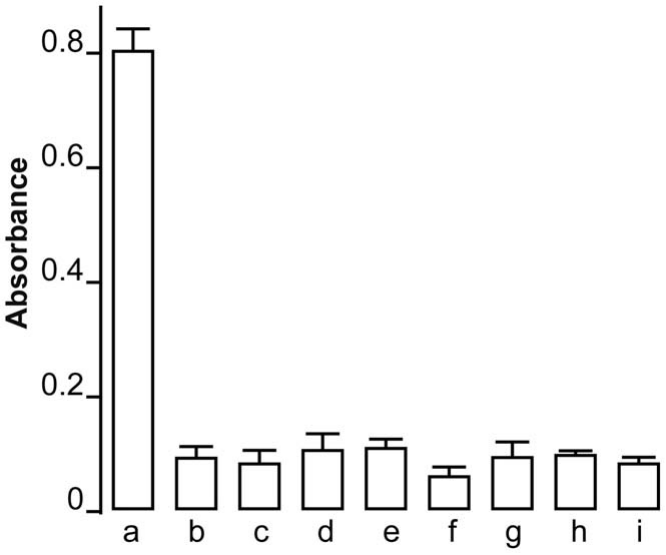
Evaluation of the presence of antibodies against Ndi1 protein in the rat serum. Rats were injected with rAAV-NDI1 either in the skeletal muscles or the brain. One group of rat received an injection of purified Ndi1 protein in the tail vein. Blood samples of each group of rats (n = 4) were drawn and the presence of anti-Ndi1 antibodies was assessed by using ELISA. A 96 wells plate was coated with purified Ndi1 protein and the sera were tested from the following groups (serum dilution in parenthesis): a, Ndi1 protein injected into tail vein (1/5000); b, PBS injected muscle, 1 week (1/100); c, rAAV-NDI1 injected muscle, 1 week (1/100); d, PBS injected muscle, 1 month (1/100); e, rAAV-NDI1 injected muscle, 1 month (1/100); f, PBS injected brain, 1 week (1/100), g; rAAV-NDI1 injected brain, 1 week (1/100); h, PBS injected brain, 1 month (1/100); i, rAAV-NDI1 injected brain 1, month (1/100). Data are presented as mean ± SD.

It is known that the GFP protein is immunogenic in higher animals [20] and could pose serious problems if introduced into the human body. Total lack of immune response in the rats expressing Ndi1 in the muscle and brain prompted us to examine if this yeast protein is actually recognized by the host animal as an antigen. To characterize the humoral immune response against Ndi1, we injected purified Ndi1 protein into the tail vein of the rats and analyzed the serum for anti-Ndi1 antibody. Blood samples were drawn from the animals and assayed for the presence of anti-Ndi1 antibody by ELISA using 96 wells immulon 2HB plates coated with purified Ndi1 protein. The antibody against Ndi1 was detected even when the serum was diluted at 1∶5000, indicating that Ndi1 is indeed highly immunogenic in rats (Figure 5). Using the same method, we next tested the sera withdrawn from the rats injected with rAAV-NDI1 in the muscle. Even at a dilution of 1∶100, no differences were noticed between the animals that were injected with PBS and those with rAAV-NDI1. Even after 6 months of Ndi1 expression, no detectable amount of anti-Ndi1 antibody was present in the rat sera. The same negative result was obtained with the rats that received rAAV-NDI1 in the brain (Figure 5).

## Discussion

It has been well established that the Ndi1 enzyme from yeast can be integrated into the oxidative phosphorylation system in the mitochondria of animals and serves as a functional replacement for complex I. Successful use of Ndi1 was reported not only for rodents [4]–[7], [15], [21] but also for other species such as *Caenorhabditis elegans* [22], *Drosophila* [17] and rabbit [23]. Long term expression of Ndi1 in the host mitochondria was indicative of no major issues with the host defense system against this foreign protein. Our earlier work on the assessment of the inflammatory potential of Ndi1 hinted that there was no positive response in the mouse brain where Ndi1 was present [15]. However, because the brain has an atypical immune system that might explain the absence of detectable immune response, it was important to test using tissues such as skeletal muscles that are abundantly encircled by the circulatory network and therefore have a high interaction with the immune system. In the current study, we were able to demonstrate no inflammatory or immune responses in rat muscles, thereby aiding in establishing a safe and effective protocol for introducing the Ndi1 enzyme in mammals.

The Ndi1 protein turned out to be highly immunogenic in rodents, which is no surprise in light of the fact that this yeast protein does not have sequence similarity with any of the known rodent proteins. Therefore the absence of immune response to the Ndi1 did not reconcile with its immunogenicity in the animals. This paradigm may well be explained by its cellular location. According to our own observations, Ndi1 expressed in the host cells, both *in vitro* and *in vivo*, is exclusively localized to mitochondria [15], [24], [25] (see also Figure S2). To be precise, we were not able to detect Ndi1 in the cytosol using the specific antibody either by histochemical analysis of the tissues or by Western blotting of the cytosolic fraction of the cells expressing Ndi1. This point was also supported by our current results using GFP. The delivery of the *GFP* gene was carried out using the same rAAV vector and the same promoter as those for the *NDI1* gene. Unlike Ndi1, however, the GFP protein was expressed in the cytosol (Figure S2) and did elicit an immune response in the host animal tissues. Our results with GFP suggested a distinct but rather mild infiltration of inflammatory cells into the muscle or brain. It was not severe enough to cause substantial damage to the tissues. Nevertheless, this development of an immune response against GFP is regarded as a potential obstacle to using this protein as a reporter molecule for gene therapy [20].

It should be noted that segregation of a protein in a subcellular compartment such as mitochondria does not totally eliminate the possibility of recognition of that protein by the immune system. For example, it was reported that peptides derived from mitochondrially encoded proteins can act as histocompatibility antigens [26]. One such protein identified was the ND1 subunit of complex I [27]. Also, antibodies against mitochondrial proteins are found in patients with certain autoimmune diseases [28]. Exactly how the mitochondrial antigens are displayed on the surface of cells is not clearly understood. In the case of Ndi1, at least in our animal experiments, no immune response was observed in the target tissues and no antibody was detected in the serum when the gene was delivered using the rAAV vector.

The sustained Ndi1 expression we have observed in rodents represents the best argument that the yeast protein does not cause immunological complications. However, striking differences of organ function and structure between rodents and human can limit the translation of the experimental results in rodents to humans. To help bridge that gap, it is necessary to conduct research using non-human primate as a next step. We have collected preliminary data using monkeys and obtained the same negative immune response (Figure S3). More future experiments should help establish the *NDI1* gene as a novel therapeutic gene with clinical potential.

## Materials and Methods

### Ethic Statement

All surgical procedures for the brain and skeletal muscle were performed in accordance with the animal care and the Scripps Research Institute animal ethic committee (08-0065-2).

### Surgical injection

Rats (Sprague-Dawley, male) were anesthetized with 3% isoflurane in O_2_ flow. In brains, injections were done into the striatum. The antero-posterior (AP) and media-lateral (ML) stereotaxic coordinates for the striatum were calculated from the bregma. The dorso-ventral (DV) coordinate was calculated from the dural surface. The injection target had the following coordinates: AP 0.2 mm; ML ±2.8 mm; DV 5 mm. Delivery of the *NDI1* gene and the *GFP* gene was carried out using serotype 5 rAAV vectors. The volume of injection was 3 µl of rAAV-NDI1 or rAAV-GFP (3.1×10^12^ genome copy/ml, Applied Viromics). In muscles, 5 µl of rAAV-NDI1 or rAAV-GFP were injected. For non-virus control, PBS of the same volume was used instead of the rAAV particles. All injections were performed on 7-week old rats.

### ELISA experiments

In order to detect antibodies generated against the Ndi1 protein, blood was drawn at the times indicated in the figure legend. ELISA was performed using immulon-2HB 96 wells plates (Thermo Scientific, MA) coated with purified Ndi1 (50 ng/well) over night at 4°C in PBS 5% fetal bovine serum (FBS). The sera were diluted at 1∶100 in PBS 10% FBS, 0.1% Tween 20 and incubated for 2 hours at ambient temperature. After washes with PBS 0.1% Tween 20, secondary anti-rat antibodies (1/3000) coupled with horse radish peroxidase were incubated for 45 minutes. Fifty-µl of Super AquaBlue ELISA substrate (eBioscience) was added to each well and absorption was monitored at 405 nm using a plate reader (Molecular Device). For a positive control, serum of rats injected with the purified Ndi1 protein was used. Briefly, at day 0, 50 µg of Ndi1 protein was mixed with 200 µl of complete Freund's adjuvant (FA) and injected subcutaneously. At days, 14, 39 and 60, 25 µg of Ndi1 mixed with incomplete FA was injected. Blood was drawn at day 71 and the serum was extracted by centrifugation.

### H&E staining

Sample sections (10 µm for the muscles and 30 µm for the brains) were stained in hematoxylin for 45 sec., rinsed in deionized water, and finally stained in eosin for 1 sec. After coloring, the sections were dehydrated through successive 3-minute ethanol bathes (70%, 95%, and 100%). A final step in xylene achieved the staining procedure. Each slide was mounted using Permount mounting medium (Fisher Scientific).

### Immunohistochemistry

For histological studies, animals were perfused with PBS followed by cold 4% (wt/vol) paraformaldehyde (pH 7.4). Skeletal muscles and brains were harvested and post-fixed for 1 hr in the same buffer and were then placed into OCT (optimum cutting temperature) compound before being frozen on dry ice. Sections were cut at 10 µm thickness for muscles and 30 µm for brains using a cryostat (Microm HM550, Thermo Scientific, MA). Immunohistochemistry for Ndi1 (1∶250), CD3 (1∶500), CD4 (1∶2000), CD8 (1∶500), CD11b (1∶500), was carried out on slide sections. The slices were blocked using Image-iT FX (Invitrogen) for 3 hours. Sections were then incubated overnight at 4°C with the primary antibodies in TBS, 10% horse serum, 0.1% triton X-100. The Ndi1 antibody was revealed with a specific horseradish peroxidase conjugated goat anti rabbit IgG (1∶1000; Calbiohem) and the TSA Plus fluorescein system kit (PerkinElmer). The CD3, CD4, CD8, CD11b antibodies were revealed with a secondary antibody Rhodamine Red-X (Molecular Probes; 1∶600). The slides were examined using a fluorescent inverted microscope (Zeiss).

### Statistical analysis

Statistical analysis of the data was performed using the Student's t-test. Results are expressed as the mean ± standard deviation (SD). Statistical significance is described in figure legend.

## Supporting Information

Figure S1 **Representative images of rat skeletal muscle sections stained for immunological markers and transgene products.** The animals received either rAAV-GFP or rAAV-NDI1 in the skeletal muscles as described in the text, and the tissue sections were subjected to immunohistochemistry either 1 week (panel A) or 1 month (panel B) after the injection. The red color represents immunostaining with the antibody against immunological marker proteins (from the top; CD3, CD4, CD8 and CD11b) and the blue color displays nuclear staining using DAPI. In each panel, the green color in the middle column is the fluorescence from GFP and the green color in the right column is immunostaining with antibody against Ndi1. In all images the red and green channels are merged.
(TIF)

Figure S2 **Cytosolic distribution of the GFP protein and localization of the Ndi1 protein in mitochondria, both expressed in the rat skeletal muscle.** The animals received either rAAV-GFP or rAAV-NDI1 in the skeletal muscles as described in the text. The tissue sections were subjected to immunohistochemical analysis. (A) The red channel represents staining for a mitochondrial marker protein. The green channel is either the green fluorescence from GFP or immunostaining with antibody against Ndi1. (B) Profiles of staining intensity of the red (mito) and the green (GFP or Ndi1) channels were plotted for a 110 µm span of the coronal muscle sections.
(TIF)

Figure S3 **Preliminary results showing lack of immune response in the monkey brain expressing yeast Ndi1.** Two squirrel monkeys (female, weighing between 0.5 and 0.6 kg) received rAAV-NDI1 in the substantia nigra (SN) of one hemisphere of the brain at the following coordinate: AnteroPosterior: +5.7 mm from bregma, Lat: +2.5 mm from bregma, DorsoVentral: –17.5 mm from the dura mater. Brain samples were collected 2 months post-administration and were subjected to histochemical analysis. In both animals, a high level of Ndi1 expression was observed in the SN. (A) H&E staining. Monkey brain slices were stained with hematoxylin and eosin. The total number of H&E-positive cells per a field of view was counted using ImageJ software and the results were compiled in a histogram. a) the injection point, b) the SN in the hemisphere that received rAAV-NDI1, c) the SN in the other hemisphere that was not injected with the virus (control). Scale bar = 100 µm. (B) Immunohistochemical staining. Monkey brain slices were stained with antibodies against Ndi1 and each of the immunological marker proteins, CD4, CD8, CD11b or CD20. Representative images were taken from areas of a needle track and the SN expressing Ndi1. Scale bar = 200 µm.
(TIF)
