# Supplementary figures and images for: No Immune Responses by the Expression of the Yeast Ndi1 Protein in Rats
Source: PLoS One. 2011 Oct 3;6(10):e25910. doi: 10.1371/journal.pone.0025910 (PMC3185062; doi:10.1371/journal.pone.0025910)

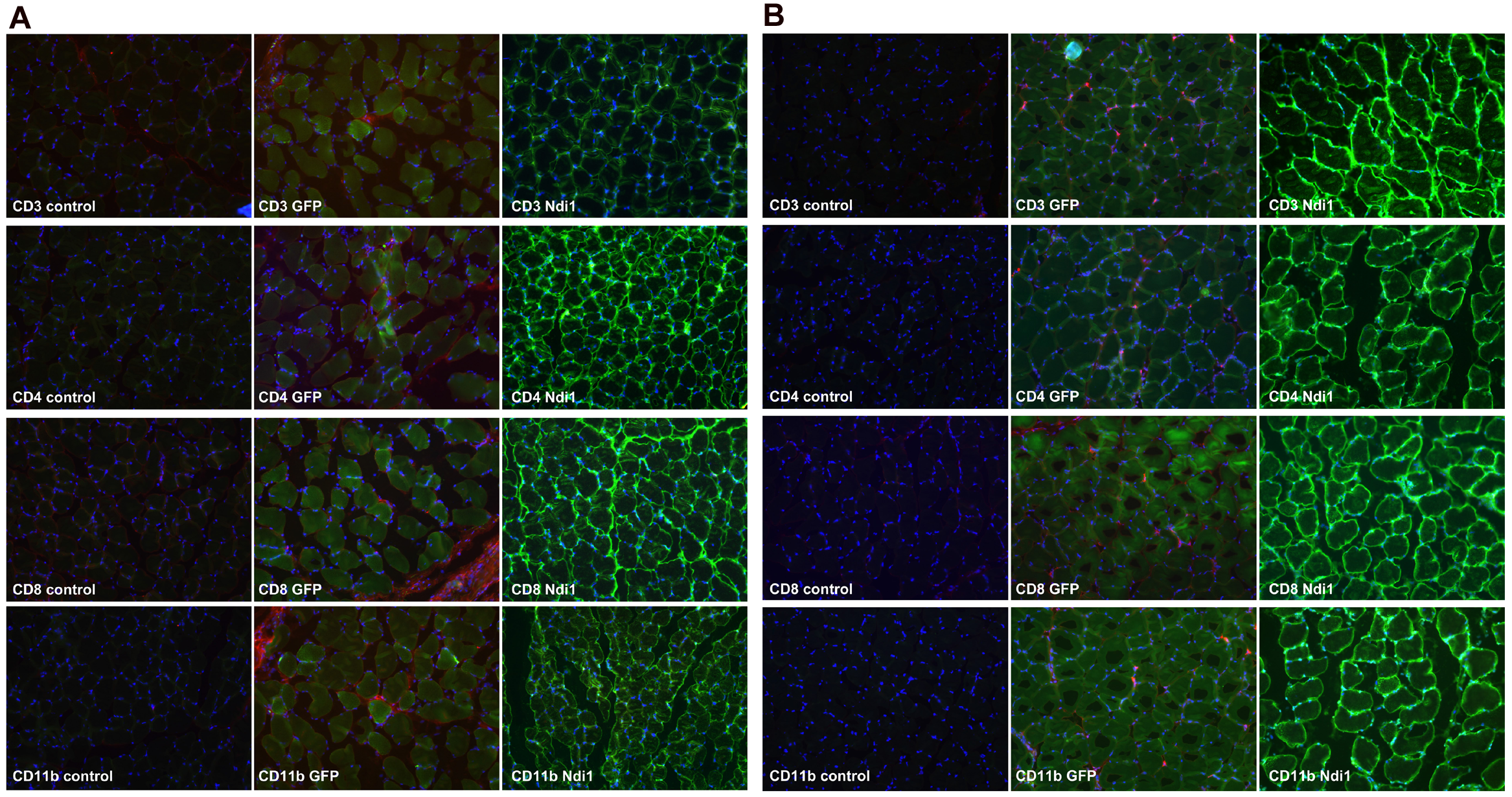

Supplement: Figure S1 — Representative images of rat skeletal muscle sections stained for immunological markers and transgene products. The animals received either rAAV-GFP or rAAV-NDI1 in the skeletal muscles as described in the text, and the tissue sections were subjected to immunohistochemistry either 1 week (panel A) or 1 month (panel B) after the injection. The red color represents immunostaining with the antibody against immunological marker proteins (from the top; CD3, CD4, CD8 and CD11b) and the blue color displays nuclear staining using DAPI. In each panel, the green color in the middle column is the fluorescence from GFP and the green color in the right column is immunostaining with antibody against Ndi1. In all images the red and green channels are merged. (TIF) [file pone.0025910.s001.tif]

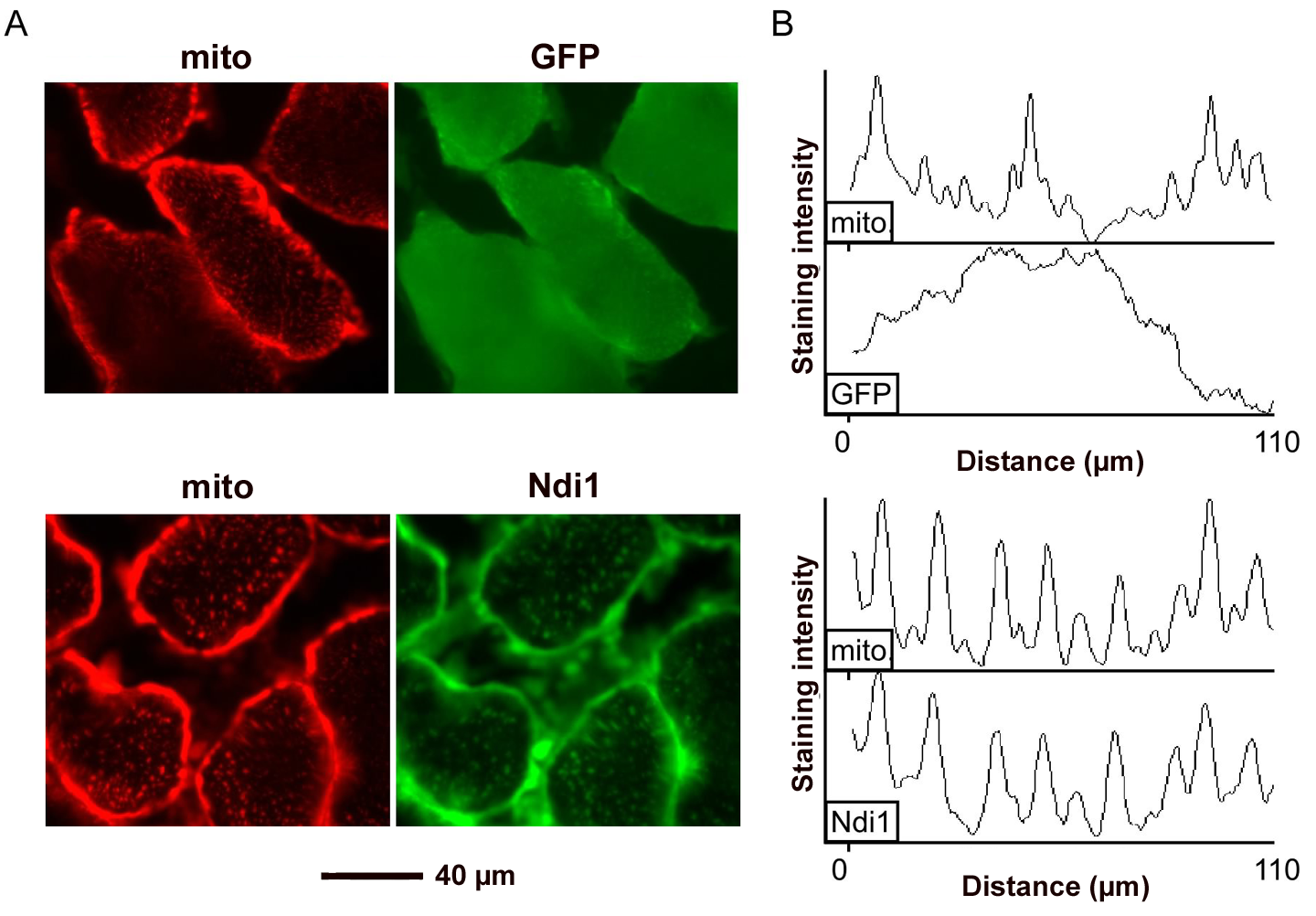

Supplement: Figure S2 — Cytosolic distribution of the GFP protein and localization of the Ndi1 protein in mitochondria, both expressed in the rat skeletal muscle. The animals received either rAAV-GFP or rAAV-NDI1 in the skeletal muscles as described in the text. The tissue sections were subjected to immunohistochemical analysis. (A) The red channel represents staining for a mitochondrial marker protein. The green channel is either the green fluorescence from GFP or immunostaining with antibody against Ndi1. (B) Profiles of staining intensity of the red (mito) and the green (GFP or Ndi1) channels were plotted for a 110 µm span of the coronal muscle sections. (TIF) [file pone.0025910.s002.tif]

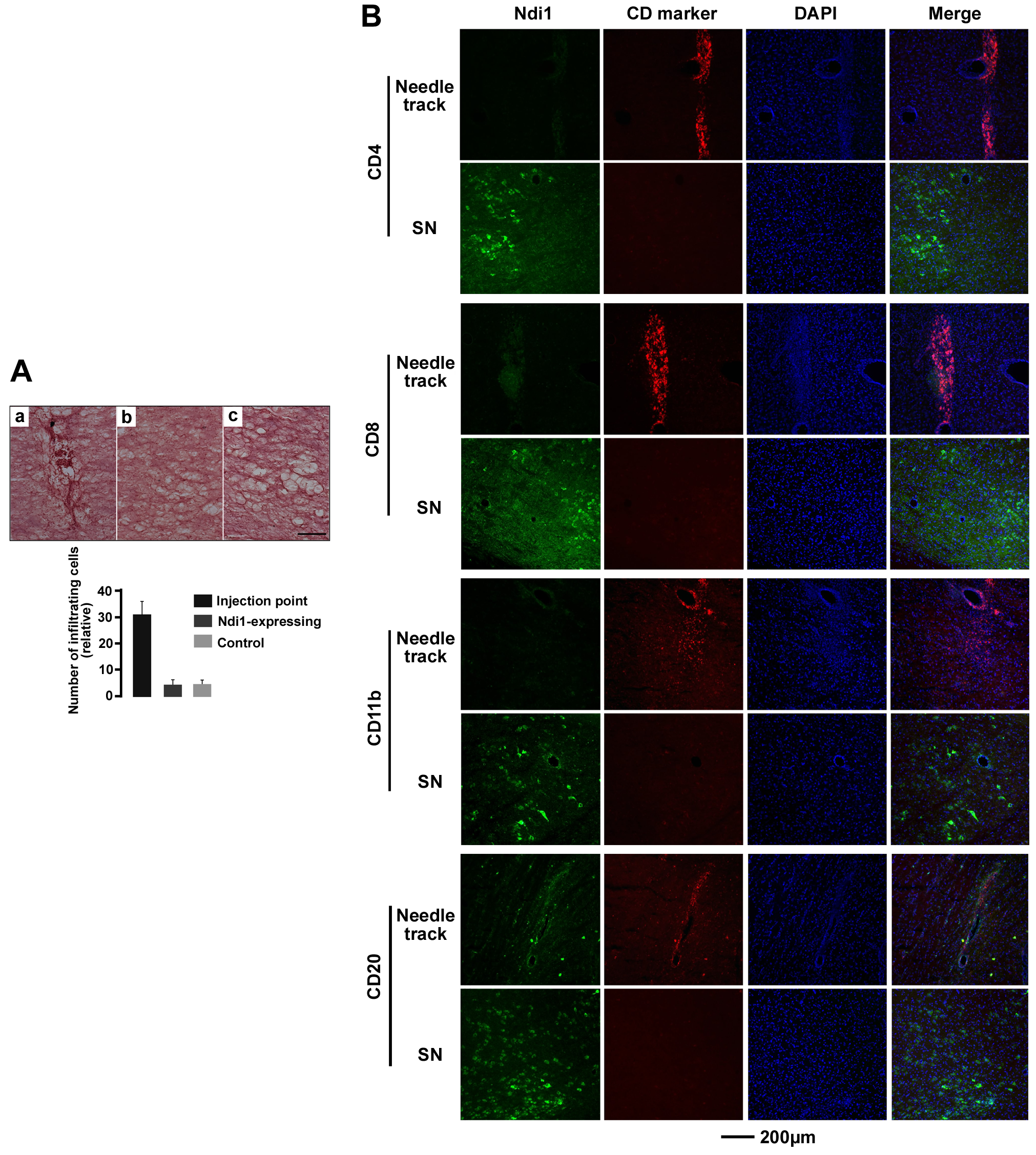

Supplement: Figure S3 — Preliminary results showing lack of immune response in the monkey brain expressing yeast Ndi1. Two squirrel monkeys (female, weighing between 0.5 and 0.6 kg) received rAAV-NDI1 in the substantia nigra (SN) of one hemisphere of the brain at the following coordinate: AnteroPosterior: +5.7 mm from bregma, Lat: +2.5 mm from bregma, DorsoVentral: –17.5 mm from the dura mater. Brain samples were collected 2 months post-administration and were subjected to histochemical analysis. In both animals, a high level of Ndi1 expression was observed in the SN. (A) H&E staining. Monkey brain slices were stained with hematoxylin and eosin. The total number of H&E-positive cells per a field of view was counted using ImageJ software and the results were compiled in a histogram. a) the injection point, b) the SN in the hemisphere that received rAAV-NDI1, c) the SN in the other hemisphere that was not injected with the virus (control). Scale bar = 100 µm. (B) Immunohistochemical staining. Monkey brain slices were stained with antibodies against Ndi1 and each of the immunological marker proteins, CD4, CD8, CD11b or CD20. Representative images were taken from areas of a needle track and the SN expressing Ndi1. Scale bar = 200 µm. (TIF) [file pone.0025910.s003.tif]
